# Safety in MR-enhanced daily adaptive SBRT Radiotherapy using a conventional C-arm linear accelerator: An FMEA approach

**DOI:** 10.1016/j.zemedi.2025.05.002

**Published:** 2025-06-03

**Authors:** Lotte Wilke, Sebastian M. Christ, Riccardo Dal Bello, Elizabeth Denney, Silvia Fabiano, Hubert S. Gabryś, Klara Kefer, Michael Mayinger, Ina Nilo, Sophie Perryck, Jens von der Grün, Matthias Guckenberger, Stephanie Tanadini-Lang

**Affiliations:** Department of Radiation Oncology, University Hospital Zurich, Switzerland

**Keywords:** SBRT, Online-adaptive RT, MR guided RT, Daily adaptive RT

## Abstract

**Background and Purpose:**

MR-guided adaptive Radiotherapy has the potential to compensate for interfractional changes in patient anatomy. Modern hybrid devices, which combine MR and linear accelerator technologies, have been clinically implemented but their costs may prevent broad adoption. To accelerate the adoption of MR-guided adaptive radiotherapy, we developed a workflow for MR-enhanced daily adaptive Radiotherapy on a C-arm linac using a dedicated MR simulator and a patient transfer shuttle system. A failure mode and effects analysis (FMEA) was performed to identify possible risks in this newly developed workflow.

**Materials and Methods:**

A workflow for MR-enhanced daily adaptive SBRT (MEDAS) on a Varian Truebeam linac was developed using a stand-alone 1.5T MR-simulator and patient transfer using a shuttle system. The different process steps were conceptualized in a multidisciplinary team and an FMEA of the different process steps was performed as well as measures for mitigation of possible risks were discussed.

**Results:**

The FMEA identified 23 failure modes across eight process steps, with the majority occurring during base plan preparation and adaptive planning. Seventeen (74%) failure modes were classified as low risk, while six (26%) were assessed as medium risk. No high-risk failure modes were identified. Risk mitigation measures, including workflow automation and checklist enhancements, successfully reduced all failure modes to low risk while not introducing new risks

**Conclusion:**

We developed a workflow for MEDAS on a conventional C-Arm linac. In this process, an FMEA was performed in a multidisciplinary team. The FMEA identified and addressed six medium-risk failure modes within the MEDAS workflow. Through further automation and adaption of existing checklists, the occurrence- and discover probability was successfully reduced, such that these failure modes are decreased to a low risk.

## Introduction

Magnetic resonance (MR)-guided online adaptive radiotherapy (MRgART) marks a significant advancement in radiation oncology, leveraging the superior soft tissue contrast of MR imaging to achieve precise tumor targeting [1].This approach allows real-time treatment adaptation, enhancing tumor dose escalation while minimizing exposure to healthy tissues, particularly in anatomically complex regions [2,3].

Traditionally, MRgART has been associated with integrated hybrid systems combining an MR scanner and linear accelerator (MR-Linac) into a single unit [4,5]. While these systems offer seamless integration, they come with significant challenges, including high costs, complex infrastructure requirements, and limited availability [6]. In contrast, a workflow employing two decoupled systems—a standalone MR scanner for imaging and a conventional C-arm linac for treatment—presents an alternative that is less resource-intensive and more cost-effective. This MR-enhanced daily adaptive SBRT (MEDAS) approach increases accessibility, enabling more healthcare facilities to offer advanced MRgART and thereby enlarge the patient population that can benefit from these innovative treatments. Feasibility of shuttle-based MR-guided RT has been investigated before by Bostel et. al. [7,8], where MR images of 20 patients were acquired prior to each treatment fraction and then compared to the cone-beam CT scans. The median time for this workflow was 61min.

The proposed decoupled MEDAS workflow involves acquiring a few selected sequences of diagnostic- MR images of the patient in treatment position on a specialized hoover table with RT immobilization devices, followed by the transportation of the patient to the linac for treatment delivery. The aim is to be finished within 1h to have a comparable treatment time as on an MR-Linac. However, this novel approach introduces unique challenges, as the workflow is not explicitly supported by vendor-designed systems. Moreover, the inherent time constraints of MEDAS—required to maintain efficient clinical operations and patient throughput—add complexity to the process.

To ensure the safe and effective implementation of this unconventional workflow, a Failure Mode and Effect Analysis (FMEA) is recommended [9,10]. FMEA is a systematic risk assessment tool that identifies potential failure modes, evaluates their impact on the process, and determines mitigation strategies to enhance safety and reliability [11,12] . Given the deviations from standard vendor protocols and the time-sensitive nature of MEDAS, conducting an FMEA is particularly critical for identifying risks [9]. By proactively addressing these risks, the clinical team can optimize the workflow, ensuring both patient safety and treatment efficacy.

This article presents a comprehensive FMEA of the proposed MEDAS workflow, offering insights into potential risks and recommendations for their mitigation. Through this analysis, we aim to establish a robust foundation for the safe adoption of decoupled MR-Linac systems in clinical practice.

## Materials and methods

### MEDAS workflow on a CBCT guided linear accelerator

A workflow for MR-enhanced daily adaptive SBRT (MEDAS) on a TrueBeam linac (MRgTB-RT) has been developed for pelvic lesions. First planning MR images are obtained from the patient using our 1.5T MR (SOLA, Siemens). The planning MRI session (30-40 min) consists of a full set of diagnostic and RT-optimized sequences, including: 3D T1 Dixon, 2D T2 turbo spin echo in each plane, 3D T1 starvibe and DWI sequences. From the 3D T1 Dixon sequence, a synthetic CT (sCT) is generated for dose calculation [[13], [14], [15]]. Patients do not require CT-appointments as the density information needed for dose calculation can be obtained from the sCT. The physician then contours the target volume(s) and organs at risk (OARs) based on the MR images and a treatment plan is calculated on the sCT. Treatment planning is performed with Eclipse treatment planning system (Varian Medical Systems, Palo Alto, USA). More details on the creation of this base plane for adaption can be found in Supplemental Material 1.

**Fig. 1:**
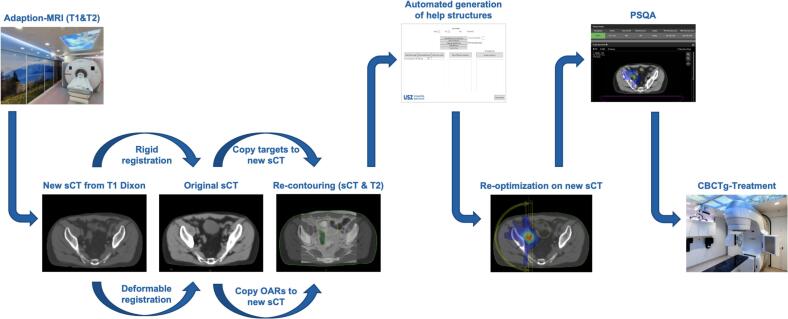
MEDAS workflow of the online adaption from the daily MRI until the treatment on a TrueBeam.

The workflow for the first treatment session is depicted in Fig. 1. A shorter MRI protocol (<10 min) including a 3D T1 Dixon and a 2D T2 turbo spin echo in the axial plan is used to obtain the daily anatomy and a new sCT is generated. The new MRI is rigidly registered with the planning MRI. Structures defined for rigid copying are then transferred to the new MRI. These are the target-volumes, nerves and bones as they are not expected to change their shape. A deformable image registration (Eclipse version 16.1, Varian Medical Systems, Palo Alto, USA) is performed between the new and original MRIs, and structures which are expected to change outline such as bowel, sigma, rectum and other OARs are deformably structures are transferred to the new MRI.

The physician then adapts the target(s) and OARs within a 2 cm ring structure around the target to match the anatomy of the day based on the T2 MRI. This is done only within the 2 cm ring to balance the time for contours adaption with the relevance of dose estimation to serial OARs. It is assumed that the 50% isodose line is fully contained within the 2 cm ring and lower doses are not relevant for serial OARs. Using an in-house developed Eclipse (Varian Medical Systems, Palo Alto, USA) scripting application programming interface (version 16.1) (ESAPI) script, any necessary help structures are created according to the rules defined during the preparation process. The original plan is then copied onto the new sCT, and the treatment-plan is re-optimized using the stored optimization template within a semi-automated workflow. If the dosimetric goals and constraints are not met, the optimization objectives may be manually adjusted until an acceptable plan is achieved. The plan is then reviewed by a physician and approved for treatment delivery.

Patient-specific secondary dose calculation with a MC based tool (SciMoCa, ScientificRT, Munic, Gerrmany) [16], MLC complexity assessment based on the mean MLC opening (MMO)[17] and a sCT QA [14,18] are subsequently conducted according to institutional guidelines. For the MC recalculation we used a gamma-evaluation with thresholds of 3%/1mm and an allowed difference in the mean PTV dose of up to 3%. For the verification of the sCT the body outline of the sCT is overridden with water density and the plan is recalculated. The mean dose in the PTV is then compared. Deviations of -1% - +4% are deemed acceptable. Once everything is ready and QA passed, the patient is transferred to the Linac using a shuttle-system (Symphony Airshuttle, CQ Medical, Greenville, USA), so that the patient can stay on the same position during MR and treatment without the need to stand-up. Treatment is delivered with CBCT image guidance. This procedure is repeated for all subsequent fractions.

### Failure mode and effect analysis

To ensure the safe implementation of this new MEDAS workflow, we performed a FMEA according to AAPM task group 100 [9]. First, we identified as many potential failure modes as possible for each process step. Then, numerical values between 1 and 10 were assigned for the likelihood of occurrence (O), severity (S), and detectability (D). These values were defined in the TG100 report. The product of these three values gives the risk priority number (RPN), which ranges from 1 to 1000. An RPN below 125 is considered low risk, between 125 and 250 medium and above high. Any high RPN must be reduced, medium RPNs should be reduced. Thes values come from our institutional experience and are used in all our FMEAs.

In a second round, any failure modes with an RPN higher than 125 were further analyzed, and possible mitigation strategies were discussed to reduce the RPN.

The FMEA was performed by a multidisciplinary expert team consisting of five medical physicists, three radiation therapists and three radiation oncologists within our department. Values for O, S and D were discussed within the team and then decided on by majority mode.

## Results

**Fig. 2:**
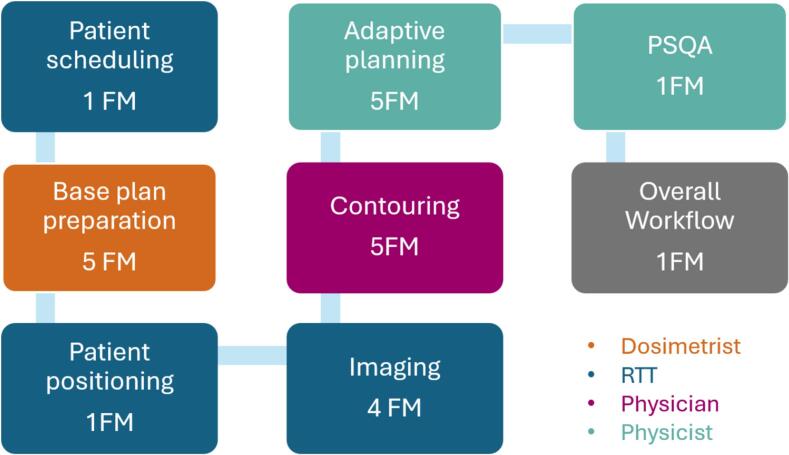
The different process steps with their number of failure modes (FM). Different colours indicate the main responsible for this step.

During the FMEA, we identified a total of 23 failure modes (FM) across eight different process steps. The different process steps and their FM are outlined in Fig. 2. The details of the FMEA are provided in the Supplemental Material 2.

**Fig. 3:**
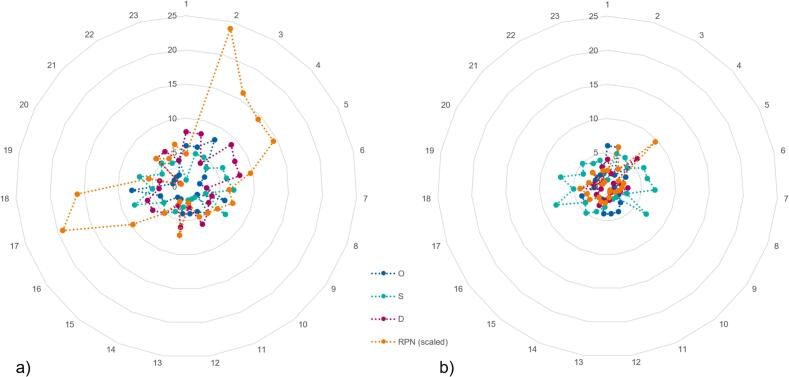
Distribution of Occurrence (O), Severity (S), Detectability (D) together with the overall Risk Priority Number (RPN). R is divided by 10 to match the scale. On the left a) before additional measures were in place and on the right b) after the additional measures were implemented.

The distribution of O, S, D, and RPN is shown in Fig. 3a. The median RPN was 63 [range 8–240]. Most of the failure modes occurred during plan preparation, contouring and adaptive planning (five each). As a result, 17 failure modes were associated with a low RPN. Six of these failure modes had an RPN of 125 or higher—four during the plan preparation step and two during adaptive planning.

To further reduce the RPN, the interdisciplinary team discussed various measures that could be implemented.

An example of an identified FM in the plan preparation was the risk that the baseplan contained to many and too complex optimization constraints. For non-adaptive treatment planning we do not have very strict guidelines for treatment planning. However, for adaptive treatment planning, it is essential that every physicist performing the adaption can quickly understand the constraints used in the baseplan to be able to adapt them if needed as otherwise it might lead to suboptimal plans in the time-pressure of the online adaption setting. Thus, we modified the checklist that during the mandatory review of the treatment plan by a physicist a check is performed ensuring standard usage of optimization constraints. One key improvement was modifying the checklist for the MRgTB-RT workflow. This updated checklist now includes detailed checks by both the planner and the reviewing physicist. The following additions were made:

• Ensuring that optimization objectives align with the standard operating procedures (SOPs) for these cases, thereby avoiding overly complex or excessive constraints that might compromise plan quality or comprehensibility.
• Verifying that correct margins are applied and confirming that the optimization process was initiated again once all optimization constraints have their final value, as changes in the constraints during the optimization could affect the plan’s integrity.
• Confirming that optimization objectives are saved in a template, which is essential for adaptive planning.

To complement the base plan checklist, we also implemented a short checklist for the adaptive process. This checklist ensures that all critical steps are covered and helps prevent severe errors. For example, it includes a step to verify that the correct MRI is used for adaptation. This is particularly important as selecting the wrong MRI during the process could easily occur.

Beyond the use of checklists, we identified automation as a critical factor in minimizing errors. For instance, margins between the clinical target volume (CTV) or gross tumor volume (GTV) and the planning target volume (PTV) are now generated using scripts. Similarly, all auxiliary structures required for planning are automatically created based on predefined rules established during base plan creation. Additionally, automated checks have been implemented to monitor changes in PTV size and assess plan complexity, ensuring consistency and accuracy throughout the process. In case the PTV size changes by more than 20% this is discussed again between physicist and physician to explain the change. Also, changes in the MMO of more than 20% need to be explained by the physicist. This should only occur in cases where for examples OARs move closer to or even inside the PTV.

While the checklists mainly improve detectability (D), the scripts primarily reduce occurrence (O).

These measures helped reduce the O and/or D values for 20 out of the 23 potential failure modes. After implementing these changes, all RPNs were below 125 (see Fig. 3b), and the median RPN decreased to 24 [6–96].

The code used ESAPI scripts is provided in a public online repository: https://github.com/medical-physics-usz/Safety-in-MR-enhanced-daily-adaptive-SBRT-Radiotherapy-using-a-conventional-C-arm-linear-accelerator.

## Discussion

The introduction of a novel workflow enabling MRgART utilizing a diagnostic MR scanner and a conventional C-arm linear accelerator necessitated a thorough evaluation to ensure its safety and efficiency. An FMEA was conducted to systematically assess the risks associated with this workflow, particularly focusing on the unique aspects introduced by the combination of these systems and the time pressure of creating treatment plans.

The FMEA methodology we employed followed the process outlined in [9], which we had successfully applied to previous implementations of innovative techniques within our clinic. Our familiarity with this approach facilitated a structured and efficient risk assessment. While other methodologies, such as the modified risk matrix used by Klüter et. al. (2021) [19] exist, we decided to follow the established guideline of the American Society of Medical Physics [9]. We believe that there is no perfect solution for a risk analysis for all possible scenarios, but it is rather important to perform one at all. A more detailed description of the advantages of different risk management processes is described by Kornek et. al. [10].

The FMEA process fostered an open, interdisciplinary discussion within our clinic, enabling team members from various specialties to contribute their perspectives. This inclusivity was particularly valuable because not all team members were involved in designing the initial workflow layout. Their fresh viewpoints helped uncover potential issues that might otherwise have been overlooked.

One major outcome of the FMEA was the improvement and adaptation of our existing checklists. We added several points specific to the online adaption into the pre-treatment checklist and developed an adaptive checklist. Both checklists are available in Supplemental Material 3. Checklists are a well-established tool in radiotherapy and other fields for mitigating errors in repetitive tasks. For example, in aviation, checklists are crucial for ensuring safety during pre-flight and emergency procedures [20]. Similarly, in surgery, safety checklists have been shown to reduce complications and improve patient outcomes [21]. By refining these tools, we aimed to minimize the risk of errors and enhance overall workflow reliability.

Additionally, the FMEA drove advancements in automation. Automation not only reduces the likelihood of human errors but also significantly accelerates processes. This is especially critical for online adaptive radiotherapy, where minimizing the time between imaging and treatment is essential for optimal patient outcomes [22]. We plan to further optimize the process automating the import, the image registration, copying of contours and automatic contouring. Other institutions have shown that the full adaptive workflow could be automized [23], however this might introduce new risks. We decided to rely on well established procedures.

The large number of FMs identified, as well as the presence of several failure modes with a RPN exceeding the threshold of 125, underscored the necessity of performing the FMEA. This analysis helped prioritize workflow optimizations and allocate resources to address the most critical steps. For new techniques where experience and guidelines are limited, conducting an FMEA is indispensable to ensuring safety and efficiency [9].

Our FMEA was conducted during the implementation phase of the new workflow. Following the publication of Kornek et al. (2024), we plan to continuously reevaluate and update the analysis based on data from our incident reporting system [24]. Currently we aim for an update every three as suggested in the DGMP report 25 and 28 [10]. This iterative approach ensures that the workflow remains robust and responsive to emerging challenges.

Limitations of this study include its conduction at a single center and the use of only one MR-to-linac combination. Additionally, not all risks can be mitigated with the scripting utilized, as it cannot modify all treatment-plan parameters. We also did not include processes which were already established in our clinic such as MRI imaging and treatment on the linacs.

In conclusion, we have successfully developed and implemented a novel workflow for online adaptive MR-guided radiotherapy using a conventional C-arm linac. To ensure patient safety and workflow efficiency, we performed a FMEA prior to the system’s go-live. This analysis identified several high-priority FMs, leading us to focus on enhancing automation and refining checklists. As we gain experience with this new technique, the workflow and associated safety measures will be updated continuously, informed by real-world feedback and incident reports. This ongoing commitment to quality improvement will ensure the safe and effective application of this advanced radiotherapy technique.

During the preparation of this work the authors used ChatGPT in order to improve readability. After using this tool, the authors reviewed and edited the content as needed and take full responsibility for the content of the publication.

## CRediT authorship contribution statement

**Lotte Wilke:** Writing – review & editing, Writing – original draft, Project administration, Investigation, Formal analysis, Data curation, Conceptualization. **Sebastian M. Christ:** Writing – review & editing, Investigation. **Riccardo Dal Bello:** Writing – review & editing, Software, Investigation, Conceptualization. **Elizabeth Denney:** Writing – review & editing, Investigation, Conceptualization. **Silvia Fabiano:** Writing – review & editing, Software, Investigation, Conceptualization. **Hubert S. Gabryś:** Writing – review & editing, Software. **Klara Kefer:** Writing – review & editing, Investigation, Conceptualization. **Michael Mayiger:** Writing – review & editing, Investigation. **Ina Nilo:** Writing – review & editing, Investigation, Conceptualization. **Sophie Perryck:** Writing – review & editing, Conceptualization. **Jens von der Grün:** Writing – review & editing, Investigation, Conceptualization. **Matthias Guckenberger:** Writing – review & editing, Supervision, Investigation, Conceptualization. **Stephanie Tanadini-Lang:** Writing – review & editing, Writing – original draft, Supervision, Methodology, Investigation, Conceptualization.

## Declaration of competing interest

The authors declare the following financial interests/personal relationships which may be considered as potential competing interests: The Department of Radiation-Oncology of the University Hospital in Zurich has research and teaching agreements with Siemens Healthineers.

Riccardo dal Bello, Michael Mayinger, Matthias Guckenberger and Stephanie Tanadini-Lang have received travel compensation from Siemens Healthineers.
