# Supplementary material for: Safety in MR-enhanced daily adaptive SBRT Radiotherapy using a conventional C-arm linear accelerator: An FMEA approach
Source: Z Med Phys. 2025 Jun 3;35(4):423–7. doi: 10.1016/j.zemedi.2025.05.002 (PMC12766485; doi:10.1016/j.zemedi.2025.05.002)
Supplement: Supplementary Data 1 [file mmc1.pdf]

Supplemental Material 1: Plan preparation process

Figure A outlines the different preparation steps for an online adaptive MR-guided treatment.

First, planning MR images are obtained from the patient using our 1.5T MR (SOLA, Siemens). These planning MR images include both T1 and T2 sequences. From the T1 sequence, a synthetic CT (sCT) is generated for dose calculation. MR and sCT is then loaded into the Eclipse Treatment planning system (Varian medical system, Palo Alto, USA, V16.1). The physician then contours the target volume(s) and organs at risk (OARs) based on the MR images. A treatment plan is created, and if any planning help-structures are required for optimization, they are generated using an in-house developed script, if applicable. The plan is then reviewed and approved by both the physician and physicist.

Next, the plan is prepared for online adaptation with the following steps:

- Detailed notes are made on potential help-structures and any compromises made due to critical OARs.
- The optimization objectives are saved.
- Two duplicates of the sCT are created: one containing structures to be rigidly copied onto the adaptation images (target volumes, nerves, bones etc.) and another for deformable structures (GI structures).
- Patient-specific quality assurance is conducted, including an independent dose calculation, validation of the sCT, and an evaluation of the plan's complexity. Depending on the complexity, portal dosimetry may be performed before the first treatment.

These steps are necessary because the planning system does not support online adaptive treatments, particularly not on an MRI.

For the MC calculation, SciMoca (ScientificRT, Munic, Gerrmany) is used [1] with a gamma-criterion of 3%/1mm larger 95% and an allowed difference in the mean PTV dose of up to 3%. For the verification of the sCT the body out-line of the sCT is overridden with water density and the plan is recalculated. The mean dose in the PTV is then compared. Deviations of -1% - +4% are deemed acceptable [2]. For the plan complexity we use the mean MLC opening (MMO) [3], where for values below 8mm a portal dosimetry has to be performed. In addition, 10% or plans are randomly drawn for portal dosimetry.

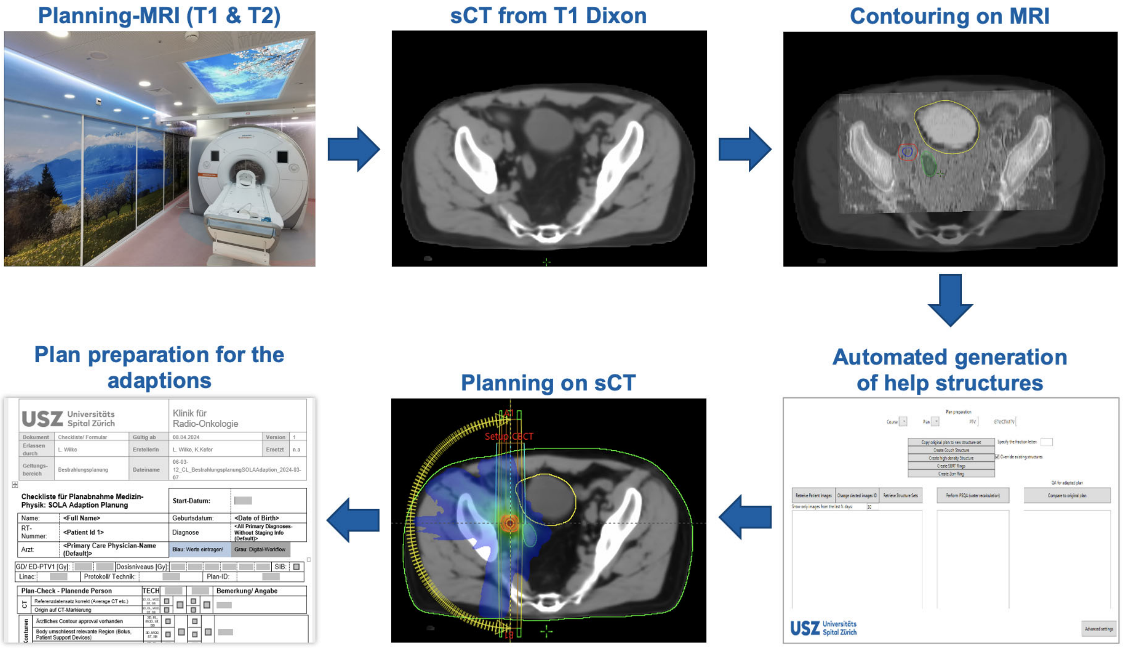

Figure A: The different steps in the plan preparation for an online adaptive treatment.

References:

1. Piffer, S., et al., *Validation of a secondary dose check tool against Monte Carlo and analytical clinical dose calculation algorithms in VMAT.* J Appl Clin Med Phys, 2021. **22**(4): p. 52-62.
2. Nella, F., S. Tanadini-Lang, and R. Dal Bello, *Clinical implementation of patient-specific quality assurance for synthetic computed tomography.* Physics and Imaging in Radiation Oncology, 2025. **34**: p. 100764.
3. Uher, K., et al., *Reduction of patient specific quality assurance through plan complexity metrics for VMAT plans with an open-source TPS script.* Z Med Phys, 2024. **34**(4): p. 555-564.
