# Supplementary material for: Safety in MR-enhanced daily adaptive SBRT Radiotherapy using a conventional C-arm linear accelerator: An FMEA approach
Source: Z Med Phys. 2025 Jun 3;35(4):423–7. doi: 10.1016/j.zemedi.2025.05.002 (PMC12766485; doi:10.1016/j.zemedi.2025.05.002)
Supplement: Supplementary Data 2 [file mmc2.pdf]

Supplemental Material 2: FMEA for online adaptive MR guided RT

					Initially				Adapded				
	**Subprocess**	**Potential failiure modes**	**Potential cause of FM**	**Effects of potential FM**	**O**	**S**	**D**	**RPN**	**new controll measure**	**O**	**S**	**D**	**RPN**
1	**Patient scheduling**	Patient was not scheduled correctly for MR and accelerator.	Incorrect scheduling	Delay in the overall program	6	1	8	48	During the release of the original plan, it is checked whether all appointments are available.	6	1	4	24
2	**Base plan preparation**	Plan is created with too many/complex constraints	Not adhering to planning guidelines	During adaptation, an adequate plan cannot be created	6	5	8	240	When releasing the original plan, a checklist is used to verify whether the optimization constraints are in accordance with the requirements	6	5	2	60
3	**Base plan preparation**	Helper structures in the original plan were not created according to the AA, so the used margins do not meet the standard	Nicht an die Vorgaben zur Planung gehalten	The plan optimization does not work correctly, no optimal plan can be created, or only after adjusting the optimization constraint	8	5	4	160	Margins are created using a script. When the plan is released, it is checked whether the margins are correct.	2	5	3	30
4	**Base plan preparation**	The last optimization was not carried out again without using the previous optimization.	No optimization from scratch in the last step.	The plan optimization is not working correctly; no optimal plan can be created, or it can only be done after adjusting the optimization constraints.	4	4	9	144	Is checked with a checklist during the planning process	4	4	6	96
5	**Base plan preparation**	The template for optimization was not saved or was saved under the wrong name.	Template for optimization not present during adaption	Plan cannot be optimized during the first adaption	3	6	8	144	Is verifyed during plan approval using the checklist.	3	6	1	18
6	**Base plan preparation**	Original structure sets/sCTs for rigid and deformable registration are not prepared.	Structure sets are not correctly created.	Poor quality of the MRI, leading to significant distortion in the treatment volume area, which can result in spatial misdosage of up to 3mm.	2	6	8	96	Is verifyed during plan approval using the checklist.	2	6	2	24
7	**Patient positioning**	Poor positioning of the patient or the coils.	Coils are not correctly placed.	Qualität des MRs nicht gut, dadurch grosse Verzeruung im Bereich des Behandlungsvolumens, dadurch räumliche Fehlbestrhalung bis 3mm möglich	3	7	3	63	Coils are only positioned after the patient is aligned with the laser; the center of the coil is then also aligned with the laser	1	7	3	21
8	**Imaging**	Part of the body outside the FOV	FOV set wrongly before scan	Body structure is incorrect, leading to inaccurate dose calculation.	6	3	4	72	The FoV is adjusted daily to match the anatomy of the day. Verification is carried out by the radiographer (MTRA) at the device before sending the MR images.	3	3	2	18
9	**Imaging**	sCT not or wrongly generated	User or algorithm error	Plan calculation on incorrectly generated sCT.	2	7	4	56	Verification of the sCT during import into Aria.	2	7	1	14
10	**Imaging**	Import of wrong images	wrong sequence sent from MR and/or imported to Eclipse	Delay in matching or contouring.	5	2	5	50	Kontrolle des Datum und Namen der Sequenz, zudem wird nur eine minimum an notwendigen Sequenzen gemacht	3	2	1	6
11	**Imaging**	Fusion incorrect	Fusion not performed incorrectly	GTV is in the wrong location; it needs to be re-contoured. Slightly altered GTV leads to longer waiting times.	4	2	6	48	The physicist performs the matching, and the physician verifies it (four-eyes principle).	4	2	1	8
12	**Contouring**	Final copy of structures to MR not done	Step in the workflow was forgotten	Incorrect structures are adjusted during contouring.	4	2	3	24	It is noted during contouring and is a point on the checklist.	4	2	2	16
13	**Contouring**	Structures are not copied correctly from original structuresets	Rules were not created during the planning process, were created incorrectly, or were created in the wrong order.	Optimization does not work as expected.	4	3	6	72	The rules are checked during the plan review, and additionally, the physicist performing the adaptation verifies the physics optimization PTV.	4	3	2	24
14	**Contouring**	CTV contouring incorrect	The physician may have contoured on the wrong sequence.	Otimization on icorrect CTV	2	4	3	24		2	4	3	24
15	**Contouring**	OAR is incorrect	Deformable registration does not work correctly, and the physician does not manually correct it.	Dose distribution not optimal	2	5	5	50	Point in Checklist to verify if OARs have been adjusted within the 2 cm ring.	2	5	2	20
16	**Contouring**	GTV slowly increasing in size	The GTV is drawn larger on the fly than it would be without time pressure.	The GTV increases from adaptation to adaptation, resulting in a larger volume being irradiated each time.	4	4	6	96	Normally, we start with the original plan, and additionally, the GTV size is compared daily with that of the original plan.	4	4	2	32
17	**Adaptive Planing**	Plan is created on wrong MRI (not the one of the day)	incorrect MRI is used	Patient wird nicht mit der korrekten Anatomie geplant	4	8	6	192	A final, independent verification to ensure the correct MR was used (checklist, possibly also in scrip).	4	8	1	32
18	**Adaptive Planing**	Auyillary structures are not or wrongly created	Incorrect margin between PTV-ITV created or couch structure forgotten.	Optimization is not working optimally.	8	5	4	160	Checklist to verify that auxiliary structures have been correctly created. Additionally, verification is done using a script script.	2	5	4	40
19	**Adaptive Planing**	Wrong optimization template is used	The wrong template was selected from the list.	Sub-optimal dose distribution	2	7	4	56	Incorrect calculations are noticed during the dose distribution review by the physicist and the physician	2	7	2	28
20	**Adaptive Planing**	Optimal dose distribution is not optained	Patient anatomy significantly different from the original plan.	Patient is not treated with the optimal plan	2	4	1	8	Any significant deterioration is re-evaluated offline to determine the cause	2	4	1	8
21	**Adaptive Planing**	Treatment with original Plan	The procedure is not followed precisely, and the necessary steps after the previous radiation are not carried out.	The old plan is accidentally not set to "early completed," resulting in radiation being delivered with the outdated plan.	2	5	6	60	Checklist and detailed work instructions.	2	5	3	30
22	**QA**	sCT QA not performed	The procedure is not followed precisely, and the sCT QA is not performed.	An incorrect sCT leads to an unacceptable deviation in dose distribution	2	4	6	48	Checklist and detailed work instructions.	2	4	1	8
23	**Overall Workflow**	Imprecise work.	Lack of attention due to the many people and the noise in the control room.		4	4	4	64	Regular reminder to remain calm.	2	4	3	24
