# Supplementary material for: Safety in MR-enhanced daily adaptive SBRT Radiotherapy using a conventional C-arm linear accelerator: An FMEA approach
Source: Z Med Phys. 2025 Jun 3;35(4):423–7. doi: 10.1016/j.zemedi.2025.05.002 (PMC12766485; doi:10.1016/j.zemedi.2025.05.002)
Supplement: Supplementary Data 3 [file mmc3.pdf]

Supplemental Material 3: Checklists

1. Checklist for the MEDAS base-plan

Changes after the FMEA are marked in red

**Checkliste für Planabnahme Medizin-Physik: MEDAS Adaption Planung**		**Start-Datum:**	
Name:	**<Full Name>**	Geburtsdatum:	**<Date of Birth>**
RT-Nummer:	**<Patient Id 1>**	Diagnose	**<All Primary Diagnoses-Without Staging Info (Default)>**
Arzt:	**<Primary Care Physician-Name (Default)>**	**Blau**: Werte eintragen!	**Grau**: Digital-Workflow

GD/ ED-PTV1 [Gy]:		Dosisniveaus [Gy]:		SIB:	☐
Linac:		Protokoll/ Technik:		Plan-ID:	

	Plan-Check - Planende Person	TECH					Bemerkung/ Angabe
CT	Referenzdatensatz korrekt (Average CT etc.)	3D, EL, MOD, ST, SB	☐	☐	☐	☐	
	Origin auf CT-Markierung	3D, EL, MOD, ST, SB	☐		☐		
Konturen	Ärztliches Contour approval vorhanden	3D, EL, MOD, ST, SB	☐	☐	☐	☐	
	Body umschliesst relevante Region (Bolus, Patient Support Devices)	3D, MOD, ST, SB	☐		☐		
	Artefakte korrigiert (Implantat, Strukturen)	3D, EL, MOD, SB	☐		☐		
Technik	Couchmodell korrekt eingefügt	3D, MOD, SB	☐	☐	☐	☐	
	Iso-Position (u.a. Lichte Weite ≥ 35 cm)	EDGE (ohne ST)	☐		☐		
	Iso – back of couch < 24cm	3D, MOD, ST, SB	☐		☐		
	Maximum zentral gelegen	SB	☐		☐		
	Jaw Tracking aktiviert	MOD, ST, SB	☐		☐		
	Luft in PTV überschrieben (Optimierung) und dann wieder nicht überschrieben (dose calc.)	MOD, ST, SB	☐		☐		
	Referenzpunkt als Primary	3D, MOD, ST, SB	☐		☐		
	Gating aktiviert	DIBH, SB Lung, Pros	☐		☐		
Adaption	PTV+2cm_Ph und Hilfsstrukturen mit MRgTB-RT_Automator Skript erstellt	Adaptive SOLA	☐	☐	☐	☐	
	Einfache Optimierung und korrekte NTO, letzte optimierung from scratch	Adaptive SOLA	☐		☐		
Plan 1+	Summenplan angelegt (S1+, Vorbelastung)	3D, EL, MOD, ST, SB	☐	☐	☐	☐	
	Vorbelastung in EQD_{2Gy} dargestellt	3D, MOD, ST, SB	☐		☐		
	Korrektes DRR fenster (Diaphragma sichtbar bei Leber?)	SB	☐		☐		
QA	Alle Felder MMO > 8mm (SBRT)<br>Alle Felder MMO > 16.5 (RA), kein PSQA	MOD,, SB	☐	☐	☐	☐	**MMO(A1)=**<br>**MMO(B1)=**
Memo	Clinical Goals added and fulfilled	3D, EL, MOD ST, SB	☐	☐	☐	☐	
	Journal Note added in ARIA (including Margins used for PTV_Ph structures)	Adaptive SOLA	☐		☐		
	Datenvalidierung (Tool) in Aria durchgeführt	3D, EL, MOD, ST, SB	☐		☐		
	Course start date correct		☐		☐		
	Leistung in Aria erfasst	3D, EL, MOD ST, SB	☐		☐		

**Datum**		**Visum**	

	Dokumentation - Planende Person	TECH					Bemerkung/ Angabe
Po[illegible]	CBCT anlegen	ST, SB	☐	☐	☐	☐	
Plan-QA	PSQA notwendig: Berechnung, Export und Linac Termin	MOD, ST, SB	☐	☐	☐	☐	
	If planned on sCT: run the PSQA script to re-calculate in water		☐		☐		
	Export nach SciMoCa	MOD, ST, SB	☐		☐		
Memo	Ärztliches Planning-Approval vorhanden	3D, EL, MOD, ST, SB	☐	☐	☐	☐	
	Plan Print Template \`USZ_standard\` sent to eDoc printer (DVH in Absolute Dose+ Couch shifts + 2D printouts there)	3D, EL, MOD, ST, SB	☐		☐		
	Machine attached and plan name added to Eingaben Task	3D, MOD, SBRT	☐		☐		
Adap-tion	Strukturenset angelegt und Strukturen kopiert	Adaption SOLA	☐	☐	☐	☐	
	Optimization objectives gespeichert	Adaptive SOLA	☐		☐		

Datum		Visum	

	S Plan-Check - Physik	TECH					Bemerkung/ Angabe
	MLC-Modulation (Jaw Tracking) oder IMRT Fluenz akzeptabel	RA,IMRT	☐	☐	☐	☐	
	Vorbelastung korrekt summiert, Summen-planäne Gewichte korrect	3D, EL, MOD ST, SB	☐	☐	☐	☐	
	Datenvalidierung in Eclipse wiederholt	3D, EL, MOD, ST, SB	☐	☐	☐	☐	
	Plan auf Reviewed gesetzt und EQD2-Course Completed?	3D, EL, MOD ST, SB	☐		☐		

Datum	Siehe Approval /	Visum	Siehe Approval /

	Plan-Check - Physik	TECH					Bemerkung/ Angabe
CT/ Konturen	Geplant auf AVE	SB	☐	☐	☐	☐	
	Body umschliesst relevante Region (Bolus, Patient Support Devices)	3D, EL, MOD ST, SB	☐		☒		
	Artefakte korrigiert (Implantat, Strukturen)	3D, MOD, SB	☐		☐		
	Couchmodell korrekt eingefügt (auf Printout)	3D, MOD, SB	☐		☐		
	Air Override-Kontur korrekt (in PTV, HU=0) und dann wieder ohne HU-Werte	MOD, SB	☐		☐		
Plan	MV6FFF/ MV10FFF	SB	☐	☐	☐	☐	
	Korrektes DRR fenster (Diaphragma sichtbar bei Leber?)	SB	☐		☐		
	Iso-Position (zur Tischkante ≤ 35 cm, zurm dorsalen Tischende ≤24cm), keine Kollosionen	3D, MOD, ST, SB	☐		☐		
Status	Ärztliches Planning-Approval vorhanden	3D, EL, MOD ST, SB	☐	☐	☐	☐	
	Geplant auf die korrekte Maschine	3D, EL, MOD ST, SB	☐		☐		
	Correct check list was chosen (MEDAS)	3D, EL, MOD ST, SB	☐		☐		
	Journal ausgefüllt	3D, EL, MOD ST, SB	☐		☐		
Plan-aus-druck	Planning-Approval Arzt auf Planausdruck	3D, EL, MOD ST, SB	☐	☐	☐	☐	
	MU Plandukument identisch zu Eclipse	3D, EL, MOD ST, SB	☐		☐		
	2D Views (axial, sag, and cor vorhanden)	3D, EL, MOD ST, SB	☐		☐		
	KISIM-Konzept entspricht Verschreibung	3D, EL, MOD, ST, SB	☐		☐		
R&V	Referenzpunkt korrekt/ Dosis Limits	3D, EL, MOD ST, SB	☐	☐	☐	☐	
	Gating aktiviert	DIBH, SB Lung, Pros	☐		☐		
QA	Datenvalidierung in Eclipse wiederholt	3D, EL, MOD, ST, SB	☐	☐	☐	☐	
	Leistung in Aria erfasst	3D, EL, MOD ST, SB	☐		☐		
	SciMoCa durchgeführt und approved	3D, MOD, ST, SB	☐		☐		
	During planfreigabe:		☐		☐		

	If planned on sCT: approve document PSQA recalculation in water (PTV Dmean within -1% - +4%)						
	Body contour on sCT checked on MR (Dixon in-phase)		☐		☐		
	Qualitätssicherung durchgeführt/geplant (Linac eingefügt)	MOD,ST,SB	☐		☐		
	Plan-Dokumente Approved	3D, EL, MOD ST, SB	☐		☐		
**Adaptionen**	PTV+2cm_Ph Kontur erstellt	Adaption SOLA	☐	☐	☐	☐	
	Strukturenset angelegt und Strukturen kopiert (Mindestens alle GTV/CTV/PTVs und für die Optimierung verwendeten!)	Adaption SOLA	☐		☐		
	Optimization template checked	Adaption SOLA	☐		☐		

**Datum**	Siehe Approval /	**Visum**	Siehe Approval /

Anleitung:

- Mit der Einzel-Check-Box nach dem Kriterium kann dieses abgewählt werden, wenn es nicht relevant ist. Bleibt die Einzel-Check-Box leer, bedeutet dies, es nicht relevant ist.
- Mit der Sammel-Check-Box wird die alle relevante Kriterien als geprüft und akzeptiert markiert. Bleibt die Sammel-Check-Box leer, bedeutet dies, es wurde ein Problem gefunden. Das Problem muss im Bemerkungsfeld erläutert werden.

1. Checklist used during the MEDAS adaption – completely new introduced after FMEA:

**Checkliste für Adaptive Workflow SOLA-TB**		**Datum:**	
Name:	**<Full Name>**	Geburtsdatum:	**<Date of Birth>**
RT-Nummer:	**<Patient Id 1>**	Diagnose	**<All Primary Diagnoses-Without Staging Info (Default)>**
Arzt:	**<Primary Care Physician-Name (Default)>**	**Blau**: Werte eintragen!	**Grau**: Digital-Workflow
Fraction#		Plan-ID:	

	Task	ARZT	PHYS	Bemerkung/ Angabe
Image Registation	Rename images (sCT and MR)		☐	
	Resample sCT to 0.1cm, width& height is multiplied by 2		☐	
	sCT length shortened to < 320 slices?		☐	
	Match R/D CT images to new MR with rigid/deformable automatic registration		☐	
	Copy structures from R/D CT to new MR		☐	
	Match original CT to new MR		☐	
Contouring	GTV/CTV adapted on MR	☐		
	OARs adapted on MR	☐		
	Margins for PTV applied on CT	☐		
	Help structures crated (see Journal note)		☐	

Manual Planning	Copy original plan to new structureset		☐	
	Use Script to generate table and help-structures		☐	
	Correct Machine selected for today?		☐	
	Load optimizing template and optimize		☐	
	Normalization correct?		☐	
	User origin correct		☐	
	Planned on MR/sCT of today?		☐	
	Plan is approved by OA	☐		
	MMO and Scimoca within limits?		☐	
	RT-Validator ok?		☐	
	Dose per fraction correct?		☐	
	Reference point same as original		☐	
	MUs within 20% from original plan		☐	
	PTV volume within 20% from original plan		☐	
	QA for sCT perforemd		☐	

**ARZT**	
**PHYS**	
